# Metagenomic Analysis of the Gastrointestinal Microbiota of *Gadus morhua callarias* L. Originating from a Chemical Munition Dump Site

**DOI:** 10.3390/toxics10050206

**Published:** 2022-04-21

**Authors:** Wojciech Wilczynski, Monika Radlinska, Klaus Wysujack, Michał Czub, Tomasz Brzeziński, Grzegorz Kowalczyk, Jacek Bełdowski, Pedro Nogueira, Piotr Maszczyk

**Affiliations:** 1 Department of Environmental Microbiology and Biotechnology, Institute of Microbiology, Faculty of Biology, University of Warsaw, I. Miecznikowa 1, 02-096 Warsaw, Poland; m.radlinska@biol.uw.edu.pl; 2 Department of Hydrobiology, Institute of Functional Biology and Ecology, Faculty of Biology, University of Warsaw, Żwirki i Wigury 101, 02-089 Warsaw, Poland; mczub@iopan.pl (M.C.); t.brzezinski@uw.edu.pl (T.B.); kowalczykgs@gmail.com (G.K.); p.maszczyk@uw.edu.pl (P.M.); 3 Thünen Institute of Fisheries Ecology, Herwigstraße 31, 27572 Bremerhaven, Germany; klaus.wysujack@thuenen.de (K.W.); pedro.nogueira@thuenen.de (P.N.); 4 Institute of Oceanology, Polish Academy of Sciences, Powstańców Warszawy 55, 81-712 Sopot, Poland; hyron@iopan.pl

**Keywords:** 16S rRNA metagenomics, eastern Baltic cod, Baltic Sea, Bornholm Deep, chemical warfare agents, CWAs, microbiome

## Abstract

Several hundred thousand tonnes of munitions containing chemical warfare agents (CWAs) are lying on the seafloor worldwide. CWAs have started leaking from corroded munitions, and their presence in the environment and in organisms inhabiting dump sites has been detected. The presence of CWAs in the water negatively affects fish, macrobenthos and free-living bacteria. It can be expected that the presence of CWAs would also affect the gut-associated bacteria in fish, which are vital for their condition. The main aim of this study was to test if the microbiota of cod collected in the Baltic Bornholm Deep (highly polluted with CWAs) is dysregulated. To investigate this, we conducted metagenomic studies based on 16S rRNA gene sequencing. We found that the microbiota of cod inhabiting the dump site was significantly less taxonomically diverse compared to those from a non-polluted reference site. Moreover, taxa associated with fish diseases (e.g., *Vibrionaceae*, *Aeromonadaceae*) were more prevalent, and probiotic taxa (e.g., *Actinobacteriota*, *Rhodobacteraceae*) were less frequent in the guts of individuals from the dump site, than those from the reference site. The differences in vulnerability of various bacterial taxa inhabiting cod gastrointestinal tracts to CWAs were hypothesised to be responsible for the observed microbiota dysregulation.

## 1. Introduction

Chemical warfare agents (CWAs) are the toxic components of chemical weapons. They include choking agents designed to impede a victim’s ability to breathe (e.g., phosgene and hydrogen cyanide), vesicant agents designed to inflict chemical burn injuries upon contact with the victim’s skin (e.g., yperite and lewisite) and nerve agents designed to fatally interfere with the victim’s nervous system (e.g., tabun and sarin). During the past century, due to the enormous military potential of CWAs, they were mass produced and often exploited in numerous international conflicts.

The mass disposal of several hundred thousand tons of unwanted, obsolete or captured chemical munitions was costly and problematic. Thus, the most common means of getting rid of unused chemical munitions was dumping them into the seas and the oceans. Sea-dumping operations took place worldwide [1]. Several dumping sites have been documented in European waters. The Baltic Sea, the Skagerrak strait, the Irish Sea and the Bay of Biscay are the areas with the largest quantities of dumped chemical munitions. In the Skagerrak Strait, at least 170,000 tons of munitions containing CWAs were dumped [2,3], and in the Baltic Sea, at least 50,000 tons were dumped [4,5].

The sea-dumping of chemical munitions continued until the early 1970s. Nowadays, they pose an immense threat to numerous aquatic ecosystems and human well-being [6,7,8]. Recent studies have reported that most shells and casings collected from the chemical warfare (CW) dump sites are corroded to such an extent that their contents (CWAs) have started to leak into the adjacent water and sediments [7,8,9] and that their concentrations in the bottom waters will peak in the next decades [6]. Newest research suggests that CWAs are highly toxic to aquatic organisms [10,11,12] and that their continuous release from the munitions deposited in the bottom waters can negatively influence the benthic biota [13,14].

The CWAs toxicity to demersal fish has long been estimated using mathematical modelling, which screened the risk profiles of various CWAs based on their chemical structure (The Ecological Structure Activity Relationships: ECOSAR) [6]. According to these estimations, the most dangerous CWAs are the organoarsenic CWAs in the bottom waters, up to 4 m from the sediments in the CW dump sites. Although the bioaccumulation potential of CWAs in the tissues of aquatic animals is rather low [15], it has recently been reported that some of the oxidation products of CWAs are bioaccumulated and biotransformed by macrobenthos and fish [14,16,17]. Several CWAs are also known to demonstrate systemic geno- and cytotoxicity [14,18,19,20]. The latest studies on the ecotoxicity of CWAs have shown the induction of severe DNA damage in the gills of macrobenthos (Blue mussel, *Mytilus trossulus*), fish from the Mediterranean Sea (Blackbelly rosefish *Helicolenus dactylopterus* and European conger *Conger conger*) and fish from the Baltic Sea (European flounder *Platichthys flesus*, Atlantic herring *Clupea harengus* and eastern Baltic cod *Gadus morhua callarias*) [14,16,21].

Besides geno- and cytotoxicity, CWAs could exert other negative effects on the aquatic organisms, i.e., the putative effect on the microbiota (microbial consortium) inhabiting the gastrointestinal (GI) tracts of fish. The composition and diversity of GI microbiota play an important role as indicators of both water contamination [22] and the gut health of sampled fish [23]. Balanced GI microbiota plays an important role in the nutrition of fish (i.e., digesting their food and synthesizing vitamins [24,25]), increasing their resistance to pathogens [26] and aiding in the intestinal regeneration by stimulating the proliferation of epithelial cells [27].

The presence of various anthropogenic pollutants in the aquatic environment, such as copper [28], lead [29] and microplastics [30,31], can cause the dysregulation (dysbiosis) of a balanced GI microbiota of fish through modifying its bacterial composition, which could, in turn, impair their performance and lower their fitness by leading to inflammation, chronic illnesses and decreased immunity to secondary infections [32]. It can be expected that the pollution of aquatic environments by CWAs could also lead to dysbiosis in the GI tracts of fish, since it has been shown that the free-living bacteria abundance, biomass and taxonomic diversity is low in CW dump sites as compared to non-contaminated sites, which results from the vulnerability of several bacterial taxa to CWAs [13,18]. The reduced abundance and diversity of free-living bacteria at dump sites are likely to constrain the potential to reinforce the GI microbiota, whereas the uptake of toxics with water and/or food could evoke selective pressure on the bacterial communities in the GI tracts of fish.

Among the fish species that are particularly threatened by CWAs is the eastern Baltic cod (*Gadus morhua callarias*, Linnaeus, 1758), a keystone species in the Baltic ecosystem and an economically important resource. The major spawning site of the vast majority of the *G. morhua callarias* population, the Bornholm Deep [33], is also one of the main dump sites of chemical munitions, where 40,000 tons of munitions containing CWAs were deposited [2]. As a demersal species, *G. morhua callarias* is especially prone to CWAs exposure: individuals of this species live in the vicinity of submerged military objects [8,34]. For this reason, cod from the Bornholm Deep have long been monitored and studied for biomarkers of CWAs exposure [35,36]. A recent study documented biotransformation and bioaccumulation of the oxidation products of several CWAs by individuals of this species [37,38].

The aim of this study was to analyze the hitherto undescribed GI microbiota of eastern Baltic cod (*G. morhua callarias*) and to compare it to the GI microbiota of cod collected at a chemical warfare dump site (the Bornholm Deep of the Baltic Sea). We tested two hypotheses: first, that the taxonomic compositions (at the phyla, families and genera levels) of the GI microbiota of cod collected at a CWAs polluted site and at reference site are different and, second, that the taxonomic diversity of the GI microbiota of cod collected at a chemical warfare dump site is altered in comparison to that of cod collected at a reference site.

## 2. Materials and Methods

### 2.1. G. morhua callarias Sampling

**Figure 1:**
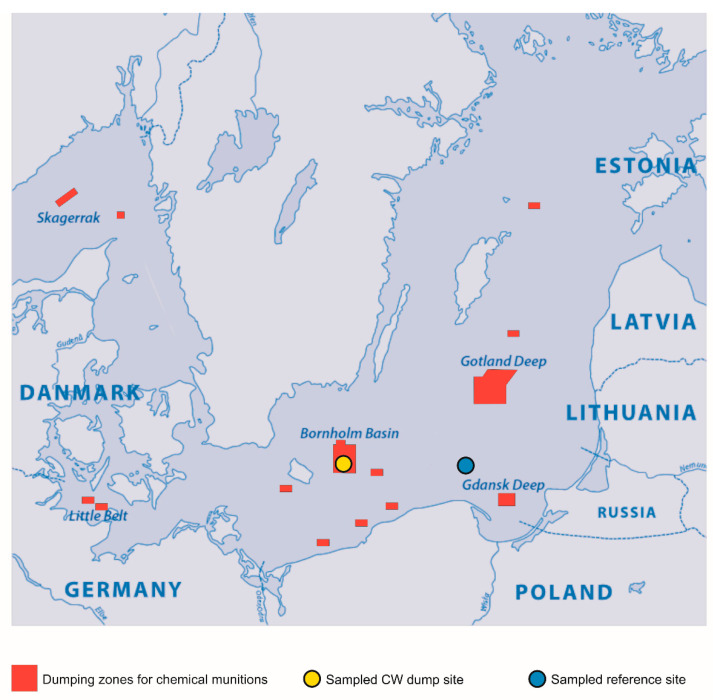
Confirmed and unconfirmed locations of deep-sea CW dump sites (red) in the Baltic Sea (reprinted with permission from [34]) with indicated sampling stations: the Bornholm Deep CW dump site (yellow circle) and the reference area (blue circle).

**Figure 2:**
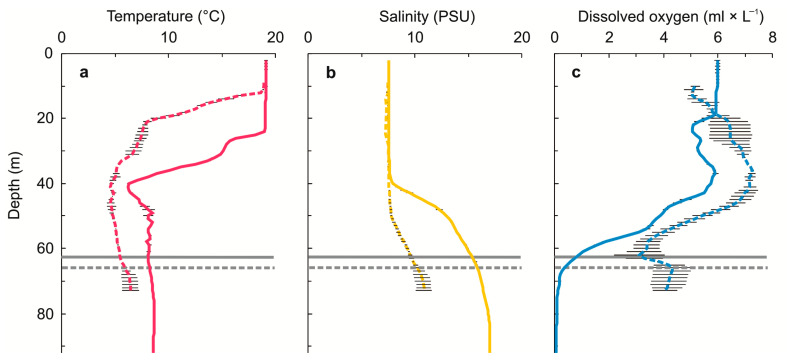
Physico–chemical parameters (± SD); temperature (**a**), salinity (**b**), dissolved oxygen (**c**) of a water column at the starting points of hauls, as well as the trawling depth at the Bornholm Deep CW dump site (solid lines) and at the reference area (dashed lines).

Specimens of *G. morhua callarias* were collected on the 18th and 19th of August 2019, using long-distance trawling during ICES monitoring cruise no. 429 of the German Fishery Research Vessel “Walther Herwig III”. The trawlings took place between 8–10 a.m local time (CEST). Two sampling sites were chosen: (1) the designated dumping area in the Bornholm Deep, around 55°18.949′N, 15°34.756′ E, where residual cod were previously confirmed to bioaccumulate CWAs-related compounds, which signifies considerable exposure to CWAs contamination [38], and (2) the DAIMON project Bornholm Deep CW dump site reference area, around 55°06.938′ N, 18°10.907′ E (Figure 1). At each site, cods were sampled from two hauls of 60 min, respectively. Among the available fish, 24 individuals were selected (10 from the reference site and 14 from the CW dump site) based solely on size similarity (28 ± 1 cm in length from the top of the mouth to the tip of the caudal fin). Such a selection decreased the risk of bias associated with size (such as different ages or diets of individuals). Before each haul, the physicochemical parameters (temperature, salinity and dissolved oxygen concentration) of the water column at the sampling stations were measured (Figure 2) using a multiparametric probe (SBE 19plus V2 SeaCAT Profiler CTD).

### 2.2. Gastrointestinal Tract and Fecal Matter Extraction

Upon capture and length measurements, individuals of adequate length were stunned by a blow on the head and afterwards put to death by severing the spinal cord and placed on ice. Next, in order to collect the GI tracts, the peritoneal cavity of each fish was aseptically opened using a scalpel, and the intestine was freed from the connective tissue, as well as adjacent internal organs. Afterwards, the esophagus was cut, and the whole GI tract (stomach, pyloric caeca and the intestine) was isolated. In order to extract its contents (fecal matter comprising of digested food and mucus secreted in the intestine), the GI tract was cut lengthwise using a same scalpel. Then, the entire contents were gently transferred to separate sterile “falcon”-type tubes (15 mL), and the samples were homogenized using a hand-held homogenizer. The samples were flash-frozen and kept at −80 °C until further processing. The whole extraction procedure was conducted in sterile conditions, using a new scalpel blade for each individual GI tract.

### 2.3. DNA Extraction and PCR Amplification

In order to isolate the total DNA from the fecal matter, the FastDNA™ Spin Kit for Feces (MP Biomedicals, Solon, OH, USA) was used, according to the manufacturer’s protocol, with 500 mg (wet mass) of each homogenized fecal matter sample representing individual fish.

The V3–V4 hypervariable region of the 16S rRNA encoding gene was amplified in the samples using universal primers 341f (5′-TCGTCGGCAGCGTCAGATGTGTATAAGAGACAGCCTACGGGnGGCwGCAG-3′) and 785r (5′-GTCTCGTGGGCTCGGAGATGTGTATAAGAGACAGGACTAChvGGGTATCTAATCC-3′), synthetized by Sigma-Aldrich, Saint Louis, Missouri, USA. The master mixes were prepared using the KAPA HiFi PCR Kit (Roche, Basel, Switzerland), and the PCR was run using the Eppendorf™ Mastercycler nexus X2 (Eppendorf, Westbury, NY, USA). The PCRs were performed in a 25 µL final reaction volume with 26 cycles of 98 °C (20 s), 55 °C (15 s) and 72 °C (15 s). The quality of DNA at each step was checked (including negative controls) by agarose gel electrophoresis and by measuring the double-stranded DNA concentration using the Qubit™ dsDNA HS Assay Kit on the Qubit™ 2.0 fluorometer (Thermo Fisher Scientific, Waltham, MA, USA). Finally, the samples containing PCR products were frozen at −20 °C until further analyses. The PCR was run in two repetitions for every individual sample while preparing new DNA template dilutions and new master mixes for each repetition. Before sequencing, two samples containing amplicons representing the microbiota of an individual GI tract (replicates) were pooled.

### 2.4. Sequencing and Data Processing

The amplicons were sequenced using an MiSeq (Illumina) platform on a single run using the MiSeq Reagent Kit v2 (Illumina, San Diego, CA, USA) and the paired-end method (2 × 300 bp), according to the standard protocols by Genomed (Warsaw, Poland).

Demultiplexing and trimming of Illumina adapter sequences (cutadapt software v3.5 [39]) was performed by the sequencing company (Genomed, Warszawa, Poland). Quality inspection, visualization and assessment of raw fastq files was performed with FastQC [40] and MultiQC [41]. The sequences were processed using the DADA2 plugin within QIIME 2 [42]. Sequences were trimmed at 270 nt, and the first 8 nt were truncated. Amplicon sequence variants (ASVs) and their counts for each sample were acquired. Alpha rarefaction plots confirmed that the number of remaining sequences was sufficient to detect the microbial diversity present. Taxonomies were assigned to the resulting ASVs with the q2-feature-classifier plugin, using the weighted Naive Bayes classifier based on the 16S rRNA silva 138 SILVA SSU gene database at 99% similarity. The Align-to-tree-mafft-fasttree pipeline from the q2-phylogeny plugin was used to construct a rooted phylogenetic tree using MAFFT. Phylogenetic and non-phylogenetic core diversity metrics, including alpha and beta diversity, were calculated using the Core-diversity-metrics pipeline. Data for this purpose were rarefied to a sampling-depth equal to the lowest frequency among the samples (23,500 reads). Phyloseq [43] and qiime2R [44] were used to plot ordination plots.

The diversity of ASVs between the samples originating from the GI tracts of cod from the reference site and the CW dump site was analyzed. Both *α* and *β* diversities, as well as the mean relative abundances of dominant ASVs, were analyzed between the studied variants. To measure *α* diversity, the Chao1 estimator and Shannon index were calculated. Chao1 is an estimator measuring the total richness, which is particularly useful because of a valid variance, which can be used to calculate associated confidence intervals [45], whereas the Shannon index reflects the species numbers and abundance equality, whereby the greater the species numbers and the evener their abundances, the higher the index value [46]. For the sake of measuring of *β* diversity, Jaccard, Bray–Curtis, unweighted UniFrac and weighted UniFrac indices were calculated. The Jaccard and unweighted UniFrac indices take into account only the number of observed ASVs, whereas the Bray–Curtis and weighted UniFrac indices consider both the number of observed ASVs and their relative abundance. The UniFrac indices additionally incorporate phylogenetic distances between observed ASVs. Basing on the mean relative abundance of ASVs in the samples, dominant phyla (top five most abundant ASVs in either group), as well as dominant families and genera (top ten most abundant ASVs in either group), were calculated. *α* diversity indices’ values were statistically compared using the Mann–Whitney U Test (statistical significance threshold was set at *p* ≤ 0.05). The bootstrap resampling method (1000 iterations) was used to calculate the confidence intervals for the differences between the mean relative abundances of dominant ASVs (an effect is statistically significant only if the corresponding confidence interval does not include zero). *β* diversity indices’ values were statistically compared using the permutational analysis of variance (PERMANOVA). The statistical analyses were performed in R Studio [47] and using the QIIME2 bioinformatics platform [42].

## 3. Results

### 3.1. Dominant ASVs

**Figure 3:**
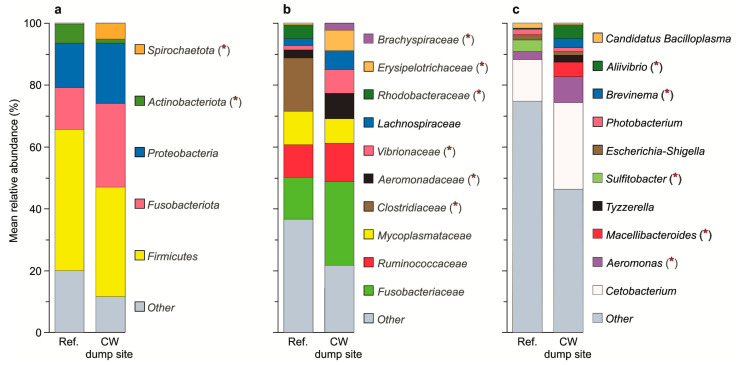
Mean relative abundances of the dominant bacteria taxa (**a**) phyla, (**b**) families and (**c**) genera (respectively, top 5, 10 and 10 most abundant ASVs in either group) and other (including unassigned) phyla, families and genera in the gastrointestinal microbiota of *G. morhua callarias* originating from either the reference site (Ref.) or the chemical warfare dump site (CW dump site). The asterisks (*) indicate statistically significant differences between the two groups.

Our results have shown that *Firmicutes*, *Fusobacteriota*, *Proteobacteria*, *Actinobacteriota* and *Spirochaetota* were the predominant bacteria in the GI tracts of the studied *G. morhua callarias* (in either group) at the level of the phylum classification. Significant differences in the mean relative abundances between the reference site and the CW dump site were observed for two phyla, *Actinobacteriota* (whose abundance was decreased) and *Spirochaetota* (whose abundance was increased), in the gut content of fish from the CW dump site (Figure 3, Table 1).

The predominant bacteria at the family level included *Fusobacteriaceae*, *Mycoplasmataceae*, *Ruminococcaceae*, *Clostridiaceae*, *Aeromonadaceae*, *Lachnospiraceae*, *Rhodobacteraceae*, *Erysipelotrichaceae*, *Brachyspiraceae* and *Vibrionaceae*. In the gut content of the fish from the CW dump site, in comparison to the reference site, *Clostridiaceae* and *Rhodobacteraceae* were significantly less abundant, while *Aeromonadaceae*, *Erysipelotrichaceae*, *Brachyspiraceae* and *Vibrionaceae* were more abundant (Figure 3, Table 1).

The most abundant genera in the GI tracts of the studied *G. morhua callarias* (in either of the two groups) comprised *Cetobacterium*, *Aeromonas*, *Macellibacteroides*, *Sulfitobacter*, *Tyzzerella*, *Escherichia*-*Shigella*, *Photobacterium*, *Brevinema*, *Aliivibrio* and *Candidatus Bacilloplasma*. Among them, the relative abundances of *Aeromonas*, *Macellibacteroides*, *Brevinema* and *Aliivibrio* were higher, while the relative abundance of *Sulfitobacter* were lower (Figure 3, Table 1), in the gut content of fish from the CW dump site, as compared to the reference site.

### 3.2. α Diversity

**Figure 4:**
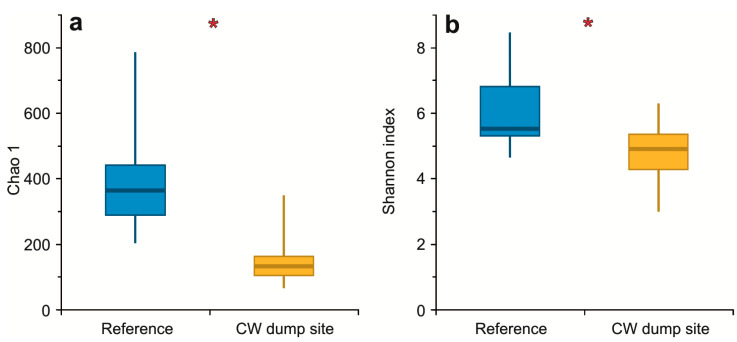
Boxplots of the (**a**) Chao 1 estimator and (**b**) Shannon index calculated on the basis of the number and the relative abundances of ASVs present in the gastrointestinal microbiota of *G. morhua callarias* from the reference site (blue) and the CW dump site (yellow). The asterisks (*) indicate statistically significant differences between the two groups.

The *α* diversity in the GI microbiota of cod originating from the CW dump site was significantly lower compared to those originating from the reference site, as evidenced by differences in the values of both the Chao 1 estimator (U = 10; Z = 3.48; *p* = 0.005) and Shannon index (U = 28; Z = 2.43; *p* = 0.015) (Figure 4).

### 3.3. β Diversity

**Figure 5:**
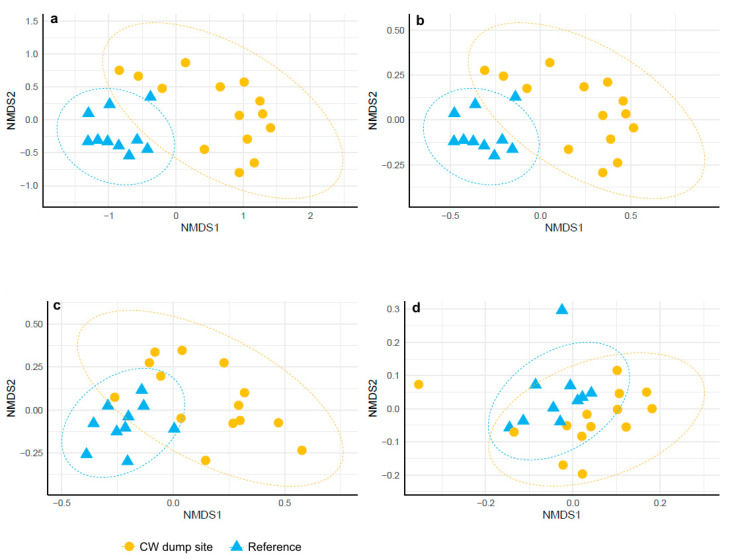
NMDS of the values of (**a**) Jaccard, (**b**) Bray–Curtis, (**c**) unweighted UniFrac and (**d**) weighted UniFrac indices calculated on the basis of the number and the relative abundances of observed ASVs and the phylogenetic distances between the observed ASVs in the gastrointestinal microbiota of *G. morhua callarias* from the reference site (blue triangles) and the CW dump site (yellow circles).

The ASVs composition in the GI microbiota of *G. morhua callarias* originating from the CW dump site was notably distinct compared to those from the reference site, as evidenced by dissimilarity indices: Jaccard (F = 0.102; *p* = 0.001), Bray–Curtis (F = 0.140; *p* = 0.001), unweighted UniFrac (F = 0.144; *p* = 0.001) and weighted UniFrac (F = 0.129; *p* = 0.002). The NMDS (Non-metric Multi-dimensional Scaling) plots revealed clear clustering of samples corresponding to the two studied groups (Figure 5).

## 4. Discussion

The metagenomic analysis, based on 16S rRNA gene sequencing, allowed the indication of the main phyla, families and genera in the GI microbiota of *G. morhua callarias* collected at the reference and the CW dump site (the Bornholm Deep). The dominant taxa observed in our study mostly reflected typical microbiota of marine fish [25,48], with especially close resemblance to the results of metagenomically analyzed microbiota of captive Atlantic cod [22].

We found significant differences between the taxonomic compositions of cod GI microbiota from the CW dump site versus reference site, which allowed us to confirm the first hypothesis of this study (that the taxonomic structure of the GI microbiota of cod collected at the two sites are different). Generally, probiotic bacteria were less abundant, and pathogenic bacteria were more abundant, in the GI tracts of cod from the CW dump site, compared with the reference site. With regard to microorganisms considered to be probiotic, the following groups of bacteria at different taxonomic levels were found to be highly reduced in the GI microbiota of cod from the CW dump site: (i) at the phylum level, *Actinobacteriota*, known for their probiotic mode of action in the guts of animals [49,50]; (ii) at the family level, *Rhodobacteraceae*, which are symbionts known for synthesizing vitamin B12 and their probiotic properties [51], and *Clostridiaceae*, solely associated with plant-based diets in fish [52,53,54]; (iii) at the genera level, *Sulfitobacter*, which comprise probiotic bacteria that are capable of inhibiting the growth of bacterial fish pathogens [55]. Simultaneously, the GI microbiota of cod from the CW dump site was characterized by a significant increase in the proportion of harmful bacteria, including the families *Aeromonadaceae, Brachyspiraceae* and *Vibrionaceae*, as well as the genera *Aeromonas, Brevinema* and *Aliivibrio*, whose various species are opportunistic pathogens associated with fish intestine diseases [56,57,58,59].

The second hypothesis of this study (that the taxonomic diversity of the GI microbiota of cod collected at the CW dump site is altered in comparison to those from the reference site) was also confirmed: the values of the *α* diversity metrics (Chao 1 estimator and Shannon index) were notably lower for the GI microbiota of cod collected at the CW dump site, as compared to cod collected at the reference site, indicating a significant decrease in the microbial diversity in the GI tracts of fish living in the vicinity of sea-dumped chemical munitions. These differences of the microbial diversity together with a significant change in the taxonomic composition of GI microbiota, with probiotic taxa diminishing and pathogenic taxa proliferating, are a symptom of dysbiosis, which is known to have severe consequences for the host. Dysregulation of the taxonomic composition of the GI microbiome in fish impairs their performance, as it is correlated with intestinal inflammation and chronic diseases [28,29,30,31].

The values of *β* diversity (the Jaccard index, the Bray–Curtis index, as well as the unweighted UniFrac and weighted UniFrac indices), indicated notable differences in the taxonomic composition (quantitative and qualitative) of the GI microbiota of *G. morhua callarias* originating from the CW dump site, as compared to those originating from the reference site. The similarity of the GI microbiota composition of cod originating from the same site and the dissimilarity of the GI microbiota composition of cod originating from different sites (in biodiversity and functional composition) additionally confirmed that the fish have been living in these sites for a time long enough to develop a site-specific microbiota, originating from adaptation to local habitats (which is the reason why the sampling took place late in the summer, long after the cod spawning season had begun).

Despite the fact that both sites were slightly different in various physico–chemical parameters (such as oxygen concentration, temperature or salinity, Figure 2), one may expect that the presence of CWAs in the dump site was one of the factors shaping the GI microbiota composition of demersal fish. Some of the *G. morhua callarias* collected from the studied dump site were earlier confirmed to come into contact with, as well as bioaccumulate, CWAs-related compounds [38]. The differences in the vulnerability of bacterial taxa to the presence of CWAs and their degradation products either dissolved in the surrounding water or bioaccumulated in the ingested food may be responsible for the observed changes in the taxonomic composition and relative abundances of GI microbiota [13]. An alternative (but not exclusive) explanation may rely on the fact that the immune system of the host also regulates the composition of the GI microbial community [60] and that CWAs and their degradation products are known to affect the immune response and condition of exposed individuals [14,18]. Exposed individuals, in a poor condition, may not be able to maintain homeostasis with their symbionts in the gut or may be more prone to infections, which also would result in changes in the GI microbiota. Although, without further studies, it is impossible to decisively distinguish whether the presence of CWAs or other environmental factors are ultimately responsible for the dysbiosis observed in the GI tracts of cod inhabiting the Bornholm deep, our results suggest that this habitat is suboptimal for adult cod.

Assuming that the results obtained in this study are associated with the presence of CWAs in one site and their absence in the other, one could expect that the CWAs would exhibit a notable selective pressure on the GI microbiota of demersal fish that are exposed to them. This would be in line with the results of previous studies, which have shown that the CWAs shape the free-living bacterial communities in the CW dump sites [13,61]. However, it should be noted that the interpretation of obtained results should be treated with caution, as the results could have been affected by the site-specific differences in the physico–chemical parameters, other pollutants unrelated to CWAs or slight differences in the diets of the studied cod besides the presence or absence of CWAs.

## 5. Conclusions

The results of this study were the first to describe the GI microbiota of eastern Baltic cod. The GI microbiota was typical for marine fish and very similar to the GI microbiota of Atlantic cod. However, the taxonomic structure of the GI microbiota of cod collected at the two studied sites was significantly distinct. The two most important differences were (1) cod from the CW dump site had significantly lower abundances of probiotic bacteria, as seen at the phylum (*Actinobacteriota*), family (*Rhodobacteraceae*) and genera (*Sulfitobacter*) levels, and (2) cod from the CW dump site had significantly higher abundances of pathogenic bacteria, as seen at the phylum (potentially, *Spirochaetota*), family (*Aeromonadaceae, Brachyspiraceae* and *Vibrionaceae*) and genus (*Aeromonas, Brevinema* and *Aliivibrio*) levels. Moreover, the GI microbiota of cods collected at the CW dump site expressed significantly reduced taxonomic diversity, as well as an overall distinct taxonomic composition (based on the bacterial number, abundance and phylogeny) when compared to those collected at the reference site.

The obtained results could become an important starting point for the future studies on the evanescing populations of this species in the Baltic Sea.

## Figures and Tables

**Table 1:** Mean relative abundances (± SD) of the dominant bacteria taxa (**a**) phyla, (**b**) families and (**c**) genera (respectively, top 5, 10 and 10 most abundant ASVs in either group) in the gastrointestinal microbiota of *G. morhua callarias* originating from either the reference site (Reference) or the chemical warfare dump site (CW dump site) and the bootstrap confidence intervals for differences in means between the two groups.

Rank	ASV	Relative Abundance (%) Mean ± SD		Difference in Means: Bootstrap Confidence Interval (95%)
		Reference	CW Dump Site	
Phylum	*Fusobacteriota*	13.5 ± 11.2	27.0 ± 24.4	−28.603~0.252
	*Firmicutes*	42.6 ± 25.1	35.5 ± 21.5	−11.048~25.784
	*Proteobacteria*	14.5 ± 12.1	19.5 ± 16.7	−16.154~6.386
	*Actinobacteriota*	6.10 ± 4.66	1.35 ± 2.20	1.844~7.914
	*Spirochaetota*	0.30 ± 0.84	5.09 ± 9.29	−10.093~−0.205
Family	*Fusobacteriaceae (Fusobacteriota)*	13.5 ± 11.2	27.0 ± 24.4	−28.007~0.472
	*Mycoplasmataceae (Actinobacteriota)*	10.7 ± 11.6	7.84 ± 9.21	−5.194~10.839
	*Ruminococcaceae (Firmicutes)*	10.6 ± 9.85	12.5 ± 9.66	−9.385~6.089
	*Clostridiaceae (Firmicutes)*	17.3 ± 23.6	0.05 ± 0.15	5.360~34.340
	*Aeromonadaceae (Proteobacteria)*	2.56 ± 1.84	8.13 ± 8.91	−10.573~−1.269
	*Lachnospiraceae (Firmicutes)*	2.26 ± 2.57	6.02 ± 8.81	−9.321~0.277
	*Rhodobacteraceae (Proteobacteria)*	4.41 ± 9.61	0.08 ± 0.13	0.962~11.383
	*Erysipelotrichaceae (Firmicutes)*	0.57 ± 0.89	6.62 ± 8.69	−10.837~−2.056
	*Brachyspiraceae (Spirichaetota)*	0.04 ± 0.10	2.27 ± 4.74	−4.730~−0.080
	*Vibrionaceae (Proteobacteria)*	1.41 ± 3.49	7.75 ± 11.1	−13.013~−0.777
Genus	*Cetobacterium * *(Fusobacteriaceae, Fusobacteriota)*	13.5 ± 11.2	27.4 ± 24.4	−29.601~0.084
	*Aeromonas* *(Aeromonadaceae, Proteobacteria)*	2.56 ± 1.84	8.19 ± 8.90	−10.296~−1.185
	*Macellibacteroides* *(Tannerellaceae, Bacteroidota)*	0.04 ± 0.07	4.60 ± 15.3	−13.713~−0.100
	*Sulfitobacter* *(Rhodobacteraceae, Proteobacteria)*	3.78 ± 9.75	0.06 ± 0.08	0.375~10.765
	*Tyzzerella* *(Lachnospiraceae, Firmicutes)*	0.00 ± 0.00	2.11 ± 7.79	0.000~6.762
	*Escherichia-Shigella* *(Enterobacteriaceae, Proteobacteria)*	1.72 ± 5.33	1.20 ± 2.72	−2.238~4.428
	*Photobacterium* *(Vibrionaceae, Proteobacteria)*	1.18 ± 3.43	3.35 ± 5.74	−5.842~1.282
	*Brevinema* *(Brevinemataceae, Spirochaetota)*	0.26 ± 0.74	2.96 ± 5.34	−5.582~−0.263
	*Aliivibrio* *(Vibrionaceae, Proteobacteria)*	0.21 ± 0.22	4.37 ± 7.65	−8.126~−0.650
	*Candidatus Bacilloplasma (Mycoplasmataceae, Firmicutes)*	1.55 ± 1.95	0.44 ± 0.98	−0.034~2.453

## Data Availability

Not applicable.
